# Drinking water quality and enteric disease: a nationwide case-crossover study (2015–2019) in New Zealand

**DOI:** 10.1038/s41370-026-00857-8

**Published:** 2026-03-25

**Authors:** Tim Chambers, Simon Hales, Farnaz Pourzand, Frank Dean, Alice Hyun Min Kim, Mario Puente-Sierra, Lukas Marek, Matt Hobbs, Michael G. Baker

**Affiliations:** 1 Ngāi Tahu Research Centre, University of Canterbury, Christchurch, New Zealand; 2 Department of Public Health, University of Otago, Wellington, New Zealand; 3 GeoHealth Laboratory, University of Canterbury, Christchurch, New Zealand; 4 College of Health, Wellbeing and Life Sciences, Sheffield Hallam University, Sheffield, UK

**Keywords:** Drinking water, New Zealand, Enteric disease, Environmental epidemiology

## Abstract

**Background:**

Despite the well-known effects of microbiological contamination of drinking water on enteric disease, there is limited epidemiological research investigating the relationship with drinking water quality direct indicators.

**Objective:**

To investigate the association between drinking water direct indicators and enteric disease.

**Methods:**

This study used a nationwide case-crossover study design of 46,020 cases of enteric disease between 2015 and 2019. Cases were successfully linked to a public water supply and exposure to E. coli, total coliforms, turbidity and free available chlorine quantified for case and control periods.

**Results:**

There were no statistically significant associations found between all enteric, bacterial-only or protozoan-only enteric disease notifications and water quality direct indicators. The presence of *E. coli* was associated with increased risk of enteric disease in supplies served by surface water (OR 1.23: 95% CI 1.04, 1.46), with known source water risks (OR 1.28: 95% CI 1.08, 1.52) and where the case was exposed to the highest tertile of rainfall (OR 1.27: 95% CI 1.01, 1.58).

**Significance:**

Major outbreaks of enteric disease can be caused by contamination of public drinking water supplies. Our findings suggest that this mechanism might also be responsible for sporadic cases of enteric disease caused by zoonotic bacterial pathogens.

**Impact:**

- This nationwide case-crossover study investigates the relationship between microbiological water quality and enteric disease in New Zealand. Linking over 46,000 cases (2015–2019) to public water supplies, we found that E. coli presence, particularly in surface water supplies with known source risks and during high rainfall, was significantly associated with increased enteric disease risk. These findings provide novel evidence that routine water quality indicators may predict sporadic enteric infections, reinforcing the need for enhanced microbial monitoring and treatment, especially in high-risk supply zones.

## Introduction

Microbiological contamination of drinking water supplies has severe implications for public health, with 505,000 deaths caused each year globally [1]. An estimated 1.7 billion people use a drinking water source that is contaminated with faeces [1]. The burden of waterborne disease in New Zealand (NZ) is largely unknown, with the latest estimate in 2006 suggesting between 18,000 and 34,000 cases of gastrointestinal illness each year [2]. Recently, NZ has experienced major waterborne outbreaks, including the Havelock North outbreak of campylobacteriosis in 2016 (with 5500 probable cases) and the Queenstown outbreak of Cryptosporidiosis in 2023 (94 cases) [3, 4].

Despite the well-known relationship between microbiological contamination of drinking water and enteric disease, there is limited empirical epidemiological research investigating the direct relationship between microbiological direct indicators in drinking water, e.g., *Escherichia coli,* abbreviated to *E. coli*, total coliforms and enteric disease risk at an individual level [5].

### Direct indicators of microbiological contamination

Direct microbiological indicators of drinking water contamination are those that attempt to measure the presence or quantity of microbial communities. The most common direct microbiological indicator used for detecting probable faecal contamination of drinking water is *E. coli*. *E. coli* is the sole microbial indicator used in the Drinking Water Standards for NZ (DWSNZ), which has a Maximum Acceptable Value (MAV) of less than 1 colony-forming unit (cfu) per 100 mL of sample [5]. Its presence in drinking water is evidence of recent faecal contamination and therefore of the possible presence of pathogens. Consequently, it is also used as an indicator of other bacterial pathogens, including Campylobacter, STEC, Salmonella, as well as protozoan pathogens such as Cryptosporidium and Giardia. However, it should be noted that in NZ, *E. coli* has been a good predictor of bacterial pathogens such as *Campylobacter* in freshwater, but not protozoan pathogens [6]. Additionally, protozoan organisms are relatively resistant to chlorine disinfection in comparison with indicators like *E. coli*, which means they may survive a disinfection process that kills the indicator organisms.

Another direct microbiological indicator is the presence of coliforms, which are a group of bacteria that belong to a single taxonomic family (Enterobacteriaceae). All coliforms (except *E. coli*) can be present in both the environment and faeces, which means their presence is not necessarily an indication of faecal contamination. However, total coliforms can be a useful indicator to signal: (1) potential pathogens in drinking water; (2) adequacy of water treatment; and (3) integrity of the distribution system [7]. In NZ, the drinking water quality assurance rule requires frequent testing for total coliforms as a microbiological indicator, but there is currently no MAV for total coliforms in the DWSNZ.

Despite the use in drinking water compliance, epidemiological research into the relationship between these direct microbial indicators and enteric disease is limited [8].

### Indirect indicators of microbiological contamination

Indirect microbiological indicators of contamination include measures that are not directly linked to health concerns but can represent changes in water quality or treatment efficacy. Turbidity and free available chlorine (FAC) are routinely used as operational proxies for short-term microbiological risk. Turbidity is associated with microbiological contamination because: (1) changes in turbidity may represent increased contamination of source waters; (2) the substances, such as soil particles and organic matter, contributing to turbidity measures can shelter pathogens; (3) water with high turbidity can interfere with treatment processes (i.e. Chlorine and ultraviolet (UV) light in particular) [9]. Rapid changes in FAC levels within a water distribution system can indicate microbiological contamination (FAC is being used up by increasing contaminants) or issues with treatment (chlorine dosage issues) [10]. However, epidemiological evidence linking these indicators to illness is mixed. A 2017 narrative review of 14 mainly time-series studies reported heterogeneous associations between turbidity and enteric disease, with larger effects in vulnerable source waters and at higher absolute turbidity (>0.5 NTU) [9]. Comparable evidence for rapid shifts in other indirect indicators (e.g., FAC, pH, temperature) remains limited.

### Environmental factors and water supply characteristics

Heavy rainfall can lead to contamination of drinking water sources. Several studies internationally have attributed large outbreaks of enteric disease to heavy rainfall events [3, 11]. However, associations between rainfall and sporadic enteric infections are less well studied, and findings are not consistent [12]. On the other hand, evidence shows more consistent positive associations between ambient air temperature and several bacterial enteric diseases, which is thought to be related to the rapid replication of bacterial pathogens in the environment, especially in food, at higher temperatures [13].

The characteristics of a water supply can also be related to enteric disease risk. In general, water supplies served by groundwater are better protected against microbial contamination than surface water due to soil layers capturing pathogens that are further eliminated by biological processes [14]. However, this protection is dependent on the depth of the bore, the permeability of the soil layers and the maintenance of the bore head [14]. The presence of livestock is also an important consideration and has been epidemiologically linked with enteric disease [15, 16]. Lastly, a number of studies have highlighted the relationships between treatment efficiencies and enteric disease risk, such as the presence of protozoa or bacterial barriers, residential disinfectant and pathogen log reduction capabilities [17]. Considering these gaps in understanding, here we investigate the association between drinking water quality direct indicators and enteric disease notifications using a case-crossover study design.

## Methods

### Population and setting

The eligible population included any person living in an area served by a Water Distribution Zone (WDZ) owned by one of the 67 Territorial Authorities (TA) between 2015 and 2019. A WDZ is an area served by a particular registered publicly-owned water supply. Our previous analysis showed people on domestic self-supplies in rural areas have a higher enteric disease rate (1046 cases of campylobacteriosis per 100,000 population) compared to public supplies in urban areas (507 cases per 100,000 population) [16]. However, we have no data on drinking water quality for people on domestic self-supplies, so they are excluded from this analysis.

Spatial data on WDZ was recently compiled into a national database, which included 629 publicly-owned WDZ in NZ serving approximately 85% of the total population (more detail is published elsewhere) [18]. The remaining 15% of the population is either on a domestic self-supply (a bore/well, rain or surface water on their property) or a small private supply (supplies serving more than one property but not owned by a TA). Neighbourhood deprivation was estimated using the NZ Deprivation Index (NZDep2013), which is a measure of socioeconomic deprivation based on nine variables available in the 2013 Census data, categorising neighbourhoods into deciles (D1–the least deprived to D10–the most deprived) based on calculated scores [19].

### Enteric disease data

We collected daily individual-level notification data from the national surveillance system for notifiable diseases from 2015 to 2019. The observation period was selected based on the availability of drinking water data. For enteric diseases such as Campylobacteriosis, Cryptosporidiosis, Giardiasis, Salmonellosis and *E. coli*, cases are legally required to be reported to the local Medical Officer of Health by laboratories and health practitioners. Notification data includes positive lab results by pathogens, demographics (age and prioritised ethnicity), neighbourhood deprivation (NZDep2013), onset date, report date and 2013 meshblock code. Each case was linked to water quality characteristics based on the 2013 meshblock code.

The report date is the date that a lab notifies the Ministry of Health (MoH) of a positive enteric disease test. This date includes multiple time lags from the date of exposure, including: the pathogen incubation period; symptom onset to presentation to a health professional; sample collection, testing and reporting. The onset date is the date the case was estimated to have started to display symptoms, as determined by a case investigator and is more relevant for our current analysis. However, the onset date is not systematically recorded in the dataset, with 38.2% of cases missing an onset date. We imputed the onset date for cases with missing values based on the median number of days difference between the report and onset day among those cases with complete data by disease type (Campylobacteriosis = 7 days; *E. coli* = 8 days; Salmonellosis = 9 days; Cryptosporidiosis = 9 days; Giardiasis = 14 days).

### Drinking water quality data

The data on water quality direct indicators used in this assessment were collected as part of an official information request by the authors to the MoH and TAs. The data included routinely collected information from compliance testing between 2015 and 2019 for publicly-owned water supplies. The direct indicators of interest included *E. coli*, total coliforms, turbidity and FAC (see Supplementary Table 1 for definitions). We used tests from treated water either at the treatment plant or in the reticulation network.

The development of the exposure assessment contained multiple steps. In brief, we compiled a record of all the relationships between treatment plants and WDZ from Taumata Arowai—the water regulator, and the MoH drinking water registers from 2006 and 2011. Treatment plants or WDZ with multiple observations per day were first averaged into a single daily value.

As treatment plants often serve more than one WDZ, each treatment plant’s test result was duplicated for each WDZ it served. There are 252 WDZ that share the identical water supply network (with exactly the same treatment plants) as one or more WDZ. These 252 WDZ fall within 84 ‘virtual’ WDZ, where the water in this WDZ is likely to be very similar to other WDZ within the same virtual zone. Consequently, when a value was available for one WDZ within a virtual zone and not another, this value was duplicated. Lastly, because WDZ measurements occur further along the distribution system than treatment plants, they are assumed to be a better reflection of the finished water being consumed by people. Consequently, when WDZ values were available on the same day as a treatment plant value, we prioritised the WDZ value. An overview of the drinking water quality dataset and number of daily values by WDZ from treatment-plant derived, virtual-zone derived and WDZ-derived approaches is provided in Supplementary Fig. 1 and Supplementary Table 2.

### Water supply characteristics data

Data on the water supply characteristics, including the type of water source (ground or surface water) and treatment methods, were retrieved by researchers through correspondence with Taumata Arowai in 2024. We assumed that these characteristics are representative of the same supplies from our exposure period (2015–2019). We classified water supply characteristics as categorical variables. When a WDZ was supplied by more than one source or treatment plant, we aggregated each measure into a WDZ-level measure. If characteristics differed between multiple sources or treatment plants, we created a new ‘mixed’ value reflecting that the WDZ has a range of values (e.g., one treatment plant has a protozoa barrier while another does not). A breakdown of the number of WDZ and population served by different water supply characteristics is provided in Supplementary Table 3.

### Weather data

We used Virtual Climate Station Network data provided by the National Institute of Water and Atmospheric Research. Station observations of maximum daily temperature and precipitation were spatially interpolated using a trivariate (elevation, latitude and longitude) thin-plate smoothing spline to a regular grid of approximately 5 × 5 km covering all of NZ (11,491 grid points). The variables were then averaged within 2013 meshblock boundaries to generate daily time series by meshblock. Data were linked to enteric disease notifications by meshblock code.

### Study design

A symmetric bi-directional case-crossover study design was used to investigate drinking water quality and its associations with notified cases of enteric disease. Case-crossover studies are appropriate when potential causes of the outcomes are acute or sudden, such as air pollution and respiratory illness [20]. heat and drownings [21]. or water contamination and enteric disease [22]. In a case-crossover study, each case serves as its own control, which also partially controls for confounding by individual-level characteristics [20]. Moreover, using control periods before and after the case onset date reduces bias from time-varying factors.

Based on our literature review [20–22]. We hypothesized that drinking water contamination increased the risk of a notifiable enteric disease within one to three weeks following exposure. Initially, we tested associations between water quality parameters and enteric disease notifications at one-, two- and three-week time lags. Initial results were similar to the alternative lag periods, but with the most consistent associations for means of explanatory variables two weeks prior to the case onset. Therefore, we chose the mean of each variable with a two-week time window for the case and control periods. Case periods were defined as the two weeks prior to estimated disease onset; control periods were defined as the two weeks before and after the case period. Data were grouped according to unique case identifiers.

### Statistical analysis

We cross-tabulated the notification data by bacterial or protozoal causes, by sociodemographic characteristics (tertile of age, tertile of neighbourhood deprivation and ethnicity), by water supply characteristics (source of drinking water, protozoa barrier presence/absence, protozoa barrier log reduction requirement) and by mean daily rainfall.

We fitted conditional logistic regression models, initially for all enteric diseases combined, with a single explanatory variable, defined as the mean of *E. coli*, total coliforms, FAC, turbidity, pH, water temperature, maximum ambient temperature or rainfall. Next, we ran models in which water quality parameters were reclassified as binary variables based on median values. This meant that *E. coli* and total coliforms were classified as present if detected on any day within each two-week exposure window and absent otherwise. Models were run separately for all enteric diseases, bacterial diseases, protozoal diseases and for individual pathogens.

We ran models restricted to subsets of water supply characteristics (source of drinking water: surface, groundwater, mixed; protozoa barrier: presence/absence; protozoa barrier log reduction: none, log-3, log-4) and socio-demographic characteristics (tertiles of age, neighbourhood deprivation and by ethnicity) to examine potential effect modification by these variables.

Based on the findings from the above analyses, we extended bivariate models for *E. coli* (the sole microbial indicator of DWSNZ) further by including the presence/absence of an additional water quality direct indicator variable (FAC, turbidity, pH, water sample temperature, maximum ambient temperature or rainfall) that may also moderate the association between *E. coli* and enteric disease. We tested for non-linear effects by dividing explanatory variables that were statistically significant predictors in the initial models (*E. coli* and rainfall) into tertiles or quintiles. Finally, we included an interaction term between the presence/absence of *E. coli* and tertiles of rainfall.

## Results

### Summary of enteric disease cases

In total, there were 55,893 enteric disease notifications (2015–2019). Of these, 46,020 (82.3%) were successfully linked to a registered water supply owned by a TA. Cases were not matched either because: (1) they are on a domestic self-supply; (2) they are on a private (non-TA) registered supply. A further 31,491 (56.3%) of cases were linked to drinking water quality results in the case period. In total, 30,858 cases (55.2%) had data for at least one direct indicator of interest for the case and control periods used in the main analysis (Table 1). A detailed description of drinking water completeness by direct indicator is available in the supplementary material (Supplementary Table 4).

**Table 1:** Overview of the cases included in the analysis by sociodemographic characteristics and key water supply characteristics.

	Overall	Campylobacteriosis	Cryptosporidiosis	*E. coli*	Giardiasis	Salmonellosis
Total	30,858	17,510	3331	2077	4765	3175
Year (%)						
2015	3219 (10.4)	1971 (11.3)	193 (5.8)	117 (5.6)	552 (11.6)	386 (12.2)
2016	3403 (11.0)	1958 (11.2)	346 (10.4)	162 (7.8)	591 (12.4)	346 (10.9)
2017	6206 (20.1)	3620 (20.7)	747 (22.4)	288 (13.9)	945 (19.8)	606 (19.1)
2018	9618 (31.2)	5440 (31.1)	1273 (38.2)	692 (33.3)	1299 (27.3)	914 (28.8)
2019	8412 (27.3)	4521 (25.8)	772 (23.2)	818 (39.4)	1378 (28.9)	923 (29.1)
Age, mean (SD)	35.7 (24.4)	39.6 (24.5)	23.0 (20.0)	36.3 (27.9)	31.8 (21.7)	33.7 (24.3)
Ethnicity (%)						
Asian	2652 (8.6)	1423 (8.1)	234 (7.0)	175 (8.4)	433 (9.1)	387 (12.2)
European	21,813 (70.7)	12,489 (71.3)	2277 (68.4)	1519 (73.1)	3388 (71.1)	2140 (67.4)
Māori	2590 (8.4)	1330 (7.6)	403 (12.1)	211 (10.2)	370 (7.8)	276 (8.7)
MELAA	400 (1.3)	164 (0.9)	40 (1.2)	39 (1.9)	115 (2.4)	42 (1.3)
Pacific	1057 (3.4)	500 (2.9)	154 (4.6)	87 (4.2)	90 (1.9)	226 (7.1)
Unknown	2346 (7.6)	1604 (9.2)	223 (6.7)	46 (2.2)	369 (7.7)	104 (3.3)
Neighbourhood deprivation (%)						
1 (least)	3970 (12.9)	2272 (13.0)	394 (11.9)	254 (12.3)	637 (13.4)	413 (13.1)
2	3712 (12.1)	2126 (12.2)	388 (11.7)	235 (11.4)	588 (12.4)	375 (11.9)
3	3517 (11.5)	2043 (11.7)	381 (11.5)	229 (11.1)	511 (10.8)	353 (11.2)
4	3308 (10.8)	1920 (11.0)	332 (10.0)	230 (11.2)	503 (10.6)	323 (10.2)
5	3165 (10.3)	1795 (10.3)	360 (10.8)	224 (10.9)	475 (10.0)	311 (9.8)
6	2989 (9.7)	1719 (9.9)	335 (10.1)	229 (11.1)	410 (8.6)	296 (9.4)
7	2849 (9.3)	1587 (9.1)	309 (9.3)	169 (8.2)	484 (10.2)	300 (9.5)
8	2656 (8.6)	1505 (8.6)	296 (8.9)	197 (9.6)	370 (7.8)	288 (9.1)
9	2585 (8.4)	1428 (8.2)	257 (7.7)	156 (7.6)	463 (9.7)	281 (8.9)
10 (most)	1959 (6.4)	1025 (5.9)	267 (8.0)	137 (6.7)	310 (6.5)	220 (7.0)
Source Type						
Ground	5815 (18.9)	3396 (19.4)	617 (18.5)	453 (21.8)	727 (15.3)	622 (19.6)
Surface	20,617 (66.8)	11,630 (66.4)	2178 (65.4)	1402 (67.5)	3345 (70.2)	2062 (65.0)
Mixed	4410 (14.3)	2476 (14.1)	533 (16.0)	221 (10.6)	690 (14.5)	490 (15.4)
Proto log reduction						
0	2112 (6.9)	1323 (7.6)	196 (5.9)	55 (2.7)	265 (5.6)	273 (8.6)
99.9%	8750 (28.5)	4897 (28.1)	987 (29.9)	635 (30.8)	1331 (28.0)	900 (28.4)
99.99%	19,810 (64.5)	11,184 (64.2)	2119 (64.1)	1374 (66.5)	3146 (66.2)	1987 (62.8)
Protozoa barrier						
No	2612 (8.5)	1589 (9.1)	240 (7.2)	114 (5.5)	342 (7.2)	327 (10.3)
Yes	25,702 (83.3)	14,444 (82.5)	2780 (83.5)	1859 (89.5)	4052 (85.1)	2567 (80.9)
mixed	2527 (8.2)	1468 (8.4)	308 (9.3)	103 (5.0)	368 (7.7)	280 (8.8)
Rainfall, mean /mm day						
Control period 1	2.98	2.93	3.27	2.85	3.02	3.00
Case period	3.00	2.98	3.23	2.91	3.05	2.98
Control period 2	2.91	2.86	3.15	2.89	2.97	2.91

### Associations between drinking water direct indicators and enteric disease

There were no statistically significant associations found between all enteric, bacterial only or protozoan only enteric disease notifications and water quality parameters as linear or as binary variables (Table 2). The highest odds ratio was observed for *E. coli* (OR 1.10: 95% CI 0.97, 1.25), which was similar for all bacterial (OR 1.09: 95% CI 0.96, 1.24) and higher for all protozoan diseases (OR 1.19: 95% CI 0.81, 1.74). No statistically significant associations were identified by individual disease.

**Table 2:** Association between drinking water determinants and enteric disease notifications.

Determinand	All enteric		All bacterial		All protozoan	
	OR	(95% CI)	OR	(95% CI)	OR	(95% CI)
*E. coli*^a^	1.10	(0.97, 1.25)	1.09	(0.96, 1.24)	1.19	(0.81, 1.74)
Total coliforms^a^	1.01	(0.97, 1.06)	1.01	(0.97, 1.06)	1.01	(0.87, 1.16)
FAC^b^	1.02	(0.94, 1.12)	1.02	(0.93, 1.12)	1.04	(0.80, 1.37)
Turbidity^b^	1.02	(0.98, 1.07)	1.04	(0.99, 1.08)	0.92	(0.82, 1.04)
pH^b^	0.97	(0.90, 1.03)	0.98	(0.91, 1.05)	0.89	(0.73, 1.09)
Water temperature^b^	1.01	(0.93, 1.09)	1.01	(0.93, 1.11)	0.92	(0.68, 1.23)

^a^Presence/absence.

^b^Above and below the median value, FAC = 0.80 mg/L, Turbidity = 0.21 NTU, pH = 7.84, Temperature = 16.6 degrees C.

In bivariate models of all enteric disease combined, where water quality indicators were coded as binary variables, associations with *E. coli* were robust to the inclusion of one other direct indicator. Associations with *E. coli* were strengthened and just statistically significant, in combination with turbidity (OR 1.20: 95% CI 1.03, 1.39) and with pH (OR 1.22: 95% CI 1.01, 1.46).

### Association between *E. coli* and enteric disease by water supply characteristics

Table 3 presents the association between the presence of *E. coli* and enteric disease by water supply characteristics. The presence of *E. coli* in supplies served by surface water was associated with a 23% increased risk of bacterial enteric disease (OR 1.23: 95% CI 1.04, 1.46). *E. coli* in supplies with a protozoa barrier was associated with a 20% increased risk of bacterial enteric disease (OR 1.20: 95% CI 1.02, 1.40), while there was a 28% increased risk in supplies requiring a 4-log reduction due to source water risks (OR 1.28: 95% CI 1.08, 1.52). There were no statistically significant associations by bacterial barrier, UV treatment, filtration, chlorination or residual disinfectant.

**Table 3:** Association between E. coli and enteric disease in separate models by water supply characteristics.

Water supply characteristic	All bacterial		All protozoan	
	OR	(95% CI)	OR	(95% CI)
**Source type**				
Groundwater	0.82	(0.58, 1.16)	1.77	(0.50, 6.24)
Surface	**1.23**	**(1.04, 1.46)**	1.16	(0.72, 1.86)
Mixed	0.97	(0.75, 1.26)	1.10	(0.52, 2.34)
**Protozoa barrier**				
No barrier	0.83	(0.56, 1.23)	1.09	(0.28, 4.27)
Barrier present	**1.20**	**(1.02, 1.40)**	1.34	(0.88, 2.05)
Mixed	0.91	(0.67, 1.23)	0.61	(0.20, 1.84)
**Protozoa log reduction**				
0-log	0.90	(0.60, 1.36)	1.38	(0.36, 5.29)
3-log (99.9%)	0.88	(0.70, 1.11)	0.83	(0.44, 1.56)
4-log (99.99%)	**1.28**	**(1.08, 1.52)**	1.51	(0.90, 2.54)

### Association between drinking water direct indicators and enteric disease by demographic characteristics and season

There were consistent positive associations between the presence of *E. coli* and bacterial disease notifications in communities on supplies requiring log-4 (99.99%) protozoan reduction due to potential source water risks (e.g., livestock) by demographic characteristics (Table 4). When *E. coli* was present, cases aged between 0 and 22 had a 44% increased risk of bacterial disease (OR 1.44: 95% CI 1.06, 1.96); those living in the least deprived areas (OR 1.36: 95% CI 1.03, 1.80) or who identified as European ethnicity (OR 1.27: 95% CI 1.05, 1.54) also had a significantly increased risk of bacterial enteric disease. E. coli presence was associated with increased risk of enteric in winter (OR 1.41: 95% CI 1.06, 1.87) but not in any other season.

**Table 4:** Association between the presence of E. coli in water suppliers with known source water risks (4-log protozoa reduction required) in separate models by demographic characteristics and season.

Characteristic	Category	OR	(95% CI)
Tertile of Age	0–22	1.44	(1.06, 1.96)
	23–48	1.19	(0.88, 1.61)
	49 *and above*	1.23	(0.92, 1.63)
Tertile of neighbourhood deprivation	*Low*	1.36	(1.03, 1.80)
	*Moderate*	1.19	(0.89, 1.60)
	*High*	1.30	(0.94, 1.81)
Ethnicity	*Asian*	1.33	(0.68, 2.62)
	*NZE*	1.27	(1.05, 1.54)
	*Māori*	1.66	(0.81, 3.40)
	*Pacific*	1.33	(0.48, 3.75)
Season	*Summer (Dec-Feb)*	0.92	(0.73, 1.17)
	*Autumn (Mar-May)*	1.17	(0.90, 1.53)
	*Winter (Jun-Aug)*	1.41	(1.06, 1.87)
	*Spring (Sep-Nov)*	1.00	(0.77, 1.30)

### Association between rainfall, *E. coli* and enteric disease risk

Supplementary Table 5 shows a statistically significant relationship between daily rainfall and enteric disease, but not for maximum daily temperature. (Table 5). presents the model results for the associations between *E. coli* in drinking water, rainfall and bacterial enteric disease for all water sources and for sources requiring 99.99% reduction of protozoa. The highest tertile of rainfall was associated with a 7% increased risk of enteric disease independent of a positive test for *E. coli* in the drinking water (OR 1.07: 95% CI 1.03, 1.11). When investigating the interaction between *E. coli* presence and rainfall, there was a 27% increase in risk when *E. coli* was present and the case was exposed to the highest tertile of rainfall (OR 1.27: 95% CI 1.01, 1.58). There were no significant associations for groundwater supplies or supplies without a defined source water risk.

**Table 5:** Associations between E. coli in drinking water and bacterial enteric disease interaction effects by tertile of rainfall.

	All water sources		Sources requiring 99.99% reduction of protozoa	
Without interaction				
Variable	OR	(95% CI)	OR	(95% CI)
*Tertile of rainfall*^a^				
Low	1.00	(ref)	1.00	(ref)
Moderate	1.02	(0.99, 1.06)	1.00	(0.95, 1.05)
High	1.07	(1.03, 1.11)	1.06	(1.01, 1.11)
*E. coli*	1.09	(0.96, 1.24)	1.28	(1.08, 1.52)
With interaction				
Low rainfall, no *E. coli*	1.00	(ref)	1.00	(ref)
Low rainfall, *E. coli* present	0.93	(0.74, 1.17)	1.21	(0.86, 1.72)
Moderate rainfall, no *E. coli*	1.02	(0.98, 1.06)	1.00	(0.95, 1.04)
Moderate rainfall, *E. coli* present	1.17	(0.97, 1.41)	1.29	(1.02, 1.63)
High rainfall, no *E. coli*	1.06	(1.02, 1.11)	1.05	(1.01, 1.11)
High rainfall, *E. coli* present	1.27	(1.01, 1.58)	1.39	(1.03, 1.88)

^a^For all water sources, average rainfall by tertile was 0.7, 2.4, 5.8 mm/day and for sources requiring 99.99% reduction of protozoa, 0.8, 2.5, 5.9 mm/day.

There were elevated risks of protozoal disease when *E. coli* was present for the subset of supplies with a protozoal barrier and for supplies requiring log-4 reduction in protozoal risk, especially when rainfall was high, but these associations had wide confidence intervals and were not statistically significant (see supplementary Table 5).

## Discussion

This study examined the association between drinking water quality direct indicators and enteric disease notifications using a case-crossover study design. There was a non-significant association between *E. coli* presence in drinking water and enteric disease risk, without accounting for water supply characteristics or case demographics. Associations between *E. coli* presence and enteric disease were stronger and statistically significant in supplies served by surface water, those that contained a protozoa barrier, had a known source water risk or experienced high rainfall in the two weeks prior to onset. In addition, central estimates were statistically significant for cases aged 0–22, of European ethnicity and living in the least deprived areas.

The effect sizes associated with *E. coli* presence in drinking and enteric diseases observed in this study (between 1.10 and 1.44) are consistent with those observed in previous studies. A 2014 meta-analysis of studies assessing the relationships between *E. coli* in drinking water and enteric disease included six studies with a pooled estimate of 1.54 (95% CI 1.37, 1.74) [8]. Within the included studies, there was wide variability between effect sizes from 1.26 to 3.86, while only one study observed a statistically significant effect (RR 1.55: 95% CI 1.36, 1.76) [23]. The heterogeneity in study approaches and results highlights the difficulty in directly assessing the relationship between *E. coli* presence in drinking water and enteric disease.

We did not detect any consistent associations for any other direct indicators tested for in this analysis. It was hypothesised that lower FAC values could have been associated with an increased risk of enteric disease due to decreased disinfectant potential, but also as an indirect indicator of contamination, as FAC is degraded by contaminants. Further, it is possible that high FAC values could be a response to a known contamination event (extreme rainfall, *E. coli* detection or treatment failure), resulting in a potential U-shape risk curve where FAC values at the extreme ends of the distribution are associated with enteric disease risk. If more data were available, we would have been able to test for these effects as well as interaction effects between direct indicators.

The increased risk of infection observed in supplies served by surface waters is consistent with previous research [14]. In general, groundwater supplies are better protected from microbial contamination due to the protective soil layers, but this is particularly true for supplies drawing from deeper aquifers with semi- to non-permeable layers [14]. The increased risk associated with having a protozoa barrier is counterintuitive and likely explained by the fact that almost all the supplies without a protozoa barrier were groundwater supplies (likely greater than 30 m deep) and so were less vulnerable to contamination. The result for those supplies requiring a 4-log protozoan reduction is consistent with literature that suggests livestock are a key source of microbial contamination [15, 16]. As detailed in the methods, water supplies with a known source water risk are required to have a 4-log reduction capability; this variable is a proxy for the risk of microbial contamination at the source. Features such as bacterial barriers, filtration and chlorination have been almost universally adopted. For these covariates, there is little differential exposure, leading to low statistical power to assess their impacts.

The association between *E. coli* presence and enteric disease was statistically significant among cases aged 0–22, of European ethnicity and those living in the lowest tertile of neighbourhood deprivation. For cases aged 0–22, it is possible that younger people are more susceptible to enteric infection and/or develop more severe symptoms that trigger presentation to a general practitioner (GP) or hospital and thus notification [24]. It is also possible that children presenting with symptoms of enteric disease are more likely to present to a GP due to parental intervention (including free healthcare for children under 14) than adults, leading to a reporting bias. It is likely that the results for cases of European ethnicity and low neighbourhood deprivation are due to reporting biases where these groups are systematically more likely to access a GP than other ethnicities and people of high neighbourhood deprivation due to access, cost and systematic racism [25, 26]. The under-presentation at GPs is supported by the opposite pattern occurring for cases presenting to hospitals by ethnicity and deprivation [27]. which suggests these demographic groups have higher rates of enteric disease, but only present when symptoms are more severe.

The positive association between rainfall and sporadic bacterial enteric infections with a time lag of two weeks was broadly consistent with previous studies [12, 28]. Although the time lags involved vary across studies. The association with rainfall was restricted to water sources at higher risk of contamination, usually due to the presence of livestock. There was a positive interaction between the presence of *E. coli* and moderate or heavy rainfall, which is consistent with rainfall leading to contamination of drinking water sources by runoff from agriculture [11]. However, observed associations with rainfall might also be related to alternative transmission pathways [13]. In addition, it is possible that associations between rainfall and enteric disease are modified by climate variables outside the temporal boundaries used in this analysis. For example, contaminants can accumulate in soils during periods of dry weather, then be flushed through at the beginning of a period of high rainfall, creating a concentration effect, which has been associated with increased risk of enteric disease [29]. Relatedly, the effect of E. coli was most pronounced in winter, suggesting there may be effect modification by season. Potential explanations for this could include (1) the longer survival times of pathogens in water of lower temperature [30]. (2) overall higher rainfall and frequency of extreme rainfall events in some areas [31]. (3) land use practices such as intensive winter grazing [32].

A key limitation of this study was the representativeness and accuracy of the sampling data. Overall, only 42% of the case and control periods had daily observations for *E. coli*. Completeness was substantially lower for other direct indicators. To increase coverage, we used sampling observations from treatment plants, which may not necessarily be representative of the water consumed at the tap, as it does not account for potential contamination post-treatment. Similarly, we also imputed values observed in one zone to other zones with the same supply structure, which implicitly assumes that these virtual zones have the same risk of contamination post-treatment. Lastly, the data included a number of features that prevented confidence in the enumerated numbers, such as presence/absence tests and bounded values (e.g., >1>240>2400, etc.). However, the limited evidence available suggests that for *E. coli,* there is not a major difference in effect size as counts increase. A pooled analysis of two studies had effect sizes of 1.54, 1.56 and 1.52 when using *E. coli* count thresholds of 1, 10 and 100, respectively [8], but this is not necessarily true for other direct indicators (e.g., FAC > 0.2).

Several elements of the enteric disease data also presented unique challenges, which created uncertainty in our analyses. First, 48% of the cases were missing a symptom onset date, which had to be imputed based on the median number of days from cases with both report and symptom onset dates by disease. This created some uncertainties in the case and control periods, leading to potential spillover effects. However, sensitivity analyses with all cases with imputed onset dates excluded produced very similar results, albeit with less statistical precision (Supplementary Table 6).

Second, our analyses included all enteric disease cases, as it was not possible to exclude cases from sources such as person-to-person transmission or food contamination. For some enteric infectious diseases, water sources typically contribute only a small proportion of total cases. Campylobacter is a good example. A recent high-quality case-control study found that 84% of cases in NZ were infected with strains attributed to a poultry source, so they are largely foodborne [33]. Disease risk was associated with using private bores or rainwater as a drinking water supply, but only for 14% of people living in rural areas. Third, notifiable diseases only represent a small and specific component of the total enteric disease burden that is known to be underreported worldwide [34]. In particular, they likely represent more severe outcomes prompting medical intervention. It is unclear whether enteric disease from water contamination is likely to produce more or less clinically severe symptoms and, therefore, more or less likely to be represented in the notifiable disease data.

There is an important limitation with generalising from this study to estimate the overall burden of enteric disease from drinking water in NZ. Registered drinking water supplies cover about 85% of the resident NZ population. The nonregistered water supplies are small, often in more rural areas and generally with far fewer barriers against contamination. Further research is needed to assess their contribution to waterborne enteric disease in NZ, which is likely to be disproportionately large and needs different interventions from the public supplies described in this paper.

Lastly, while it was useful to have data on water supply characteristics, which showed some statistically significant findings, these have their own limitations. First, the data on water supply characteristics were retrieved based on the current (2024) status of that water supply. Thus, we assumed no substantial upgrades in water treatment from the observation period 2015–2019. Second, for many water supply characteristics, there was insufficient differential exposure with protozoa and bacterial barriers and residual disinfectant was almost universally implemented. Third, these characteristics were essentially treated as a presence/absence variable and do not account for the performance of those treatment methods. In addition, there are likely substantial differences in the efficacy (e.g., log-reduction) and performance (e.g., compliance) of a particular water supply characteristic, such as UV treatment.

## Conclusions

It is well established that major outbreaks of enteric disease can be caused by contamination of public drinking water supplies. Our findings suggest that in NZ, especially where water sources are at risk of contamination by livestock, sporadic cases of enteric disease caused by zoonotic bacterial pathogens may also be transmitted via drinking water.

## Supplementary information

Supplementary Information

## Data Availability

The authors are prohibited from making the data set publicly available; however, study proposals may be submitted to the New Zealand Institute for Public Health and Forensic Science (PHF Science) for access to the enteric disease surveillance data.
