# Supplementary material for: Drinking water quality and enteric disease: a nationwide case-crossover study (2015–2019) in New Zealand
Source: J Expo Sci Environ Epidemiol. 2026 Mar 25;36(4):768–76. doi: 10.1038/s41370-026-00857-8 (PMC13331738; doi:10.1038/s41370-026-00857-8)
Supplement: Supplementary file 1 — Supplementary Information [file 41370_2026_857_MOESM1_ESM.docx]

Contents

[Supplementary Table 1. Definitions of direct indicators used in this study 2](#_Toc211843531)

[Supplementary Figure 1. A schematic of values derived from treatment plant, virtual and zones. 3](#_Toc211843532)

[Supplementary Table 2. Overview of the drinking water quality dataset of daily values at the WDZ-level by direct indicator and source (derived from treatment plant value, virtual zone or directly from WDZ) 4](#_Toc211843533)

[Supplementary Table 3. Overview of the drinking water supply characteristics 5](#_Toc211843534)

[Supplementary Table 4. Drinking water data completeness by determinant in case period, case and any control period, and in the selected case and control periods. 7](#_Toc211843535)

[Supplementary Table 5. Association between maximum daily ambient temperature and mean daily rainfall and enteric disease. 8](#_Toc211843536)

[Supplementary Table 6. Association between E. coli and enteric disease in separate models by water supply characteristics (cases with imputed onset dates removed) 9](#_Toc211843537)

[Supplementary Table 7. Association between the presence of E. coli in water suppliers with known source water risks (4-log protozoa reduction required) by age in quintile groupings 10](#_Toc211843538)

# Supplementary Table 1. Definitions of direct indicators used in this study

| **Direct indicator** | **Description** | **Source** |
| --- | --- | --- |
| Escherichia coli or E. coli | A bacteria species used as an indicator of faecal contamination of water. The presence of E. coli almost certainly indicates pathogens harmful to human health are present.  **MAV: Less than 1 in 100 mL of sample** | <https://www.taumataarowai.govt.nz/assets/Uploads/Rules-and-standards/Drinking-Water-Quality-Assurance-Rules-2022-Released-25-July-2022.pdf>  <https://www.legislation.govt.nz/regulation/public/2022/0168/latest/whole.html> |
| Total coliforms | Genera in the family Enterobacteriaceae that will grow on a specific selective medium when incubated at 35°C ± 0.2°C. | https://www.taumataarowai.govt.nz/assets/Uploads/Rules-and-standards/Drinking-Water-Quality-Assurance-Rules-2022-Released-25-July-2022.pdf |
| Free Available Chlorine or FAC | The chlorine present in chlorinated water in the form of hypochlorous acid and hypochlorite ion. | https://www.taumataarowai.govt.nz/assets/Uploads/Rules-and-standards/Drinking-Water-Quality-Assurance-Rules-2022-Released-25-July-2022.pdf |
| pH | The pH scale measures how acidic or alkaline a substance is. The scale ranges from 0 to 14. A pH of 7 is neutral. A pH less than 7 is acidic, and a pH greater than 7 is basic. Pure water is neutral with a pH of 7. When chemicals are mixed with water, the mixture can become some level of either acidic or alkaline. | https://www.epa.gov/goldkingmine/what-ph |
| Turbidity | A measure of the suspended particles in a sample that causes loss of clarity by scattering light. | https://www.taumataarowai.govt.nz/assets/Uploads/Rules-and-standards/Drinking-Water-Quality-Assurance-Rules-2022-Released-25-July-2022.pdf |

# Supplementary Figure 1. A schematic of values derived from treatment plant, virtual and zones.


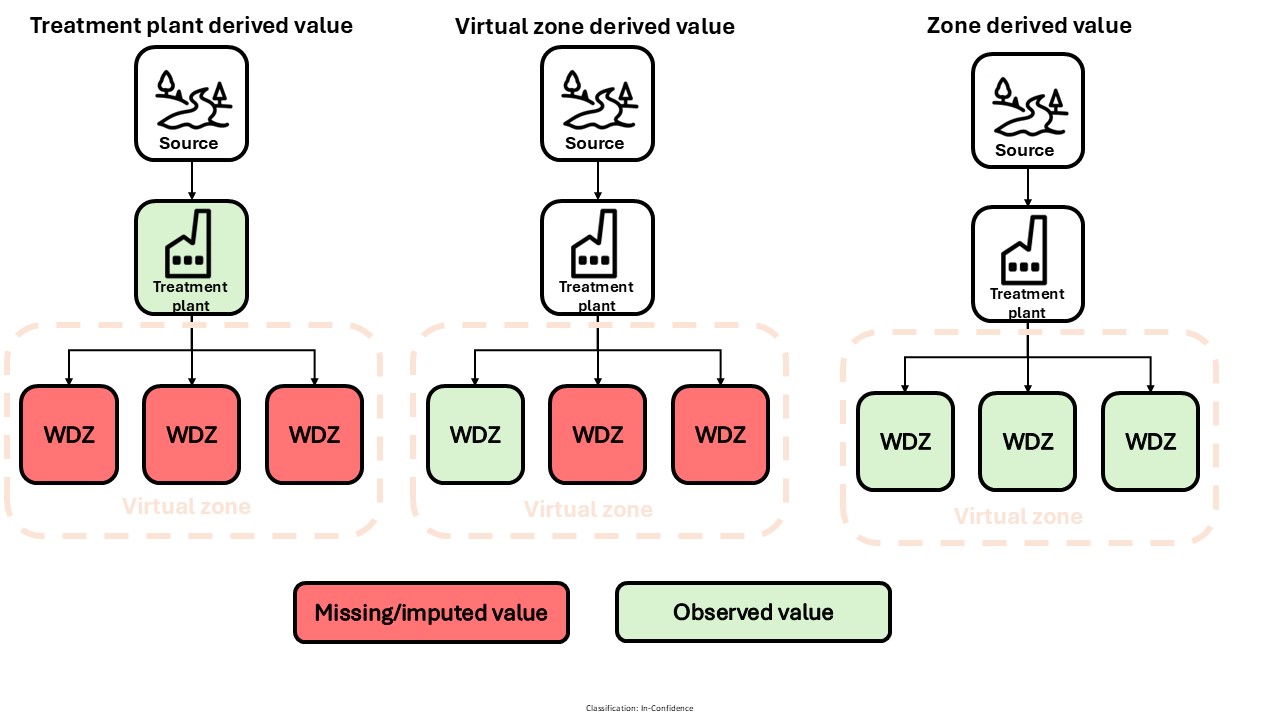


# Supplementary Table 2. Overview of the drinking water quality dataset of daily values at the WDZ-level by direct indicator and source (derived from treatment plant value, virtual zone or directly from WDZ)

An overview of the total number of observations used in the cleaned drinking water quality dataset of daily values by direct indicators and values derived by the treatment plant, WDZ and the virtual zone is presented in Supplementary Table 2. Overall, there were 836,047 daily WDZ-level observations included in the dataset from 2015–2019 which included 29.3% values derived entirely from treatment plant observations, 24.2% derived from a virtual zone (e.g. from another zone with the same treated water) and 46.6% directly observed from the WDZ. Overall, *E. coli* made up 25.0% of all direct indicator testing results, followed by total coliforms (21.2%), turbidity (17.8%), pH (16.4%), FAC (14.8%) and temperature (4.8%). It is important to note that the current testing results do not include data from known waterborne outbreaks (e.g. Havelock North).

| **Direct indicator** | **Treatment plant-derived** | | **Virtual zone-derived** | | **WDZ-derived** | | **Final dataset** |
| --- | --- | --- | --- | --- | --- | --- | --- |
|  | **n** | **%** | **n** | **%** | **n** | **%** | **n** |
| Total | 244,594 | 29.3% | 202,209 | 24.2% | 389,244 | 46.6% | 836,047 |
| **Direct measures** |  |  |  |  |  |  |  |
| *E. coli* | 74,773 | 35.8% | 30,518 | 14.6% | 103,576 | 49.6% | 208,867 |
| Total coliforms | 52,377 | 29.5% | 39,904 | 22.5% | 85,188 | 48.0% | 177,469 |
| **Indirect measures** |  |  |  |  |  |  |  |
| Turbidity | 38,672 | 26.0% | 47,396 | 31.8% | 62,875 | 42.2% | 148,943 |
| FAC | 35,109 | 28.4% | 26,393 | 21.4% | 62,052 | 50.2% | 123,554 |
| pH | 34,674 | 25.3% | 45,549 | 33.3% | 56,566 | 41.4% | 136,789 |
| Temperature | 8,989 | 22.2% | 12,449 | 30.8% | 18,987 | 47.0% | 40,425 |

# Supplementary Table 3. Overview of the drinking water supply characteristics

Supplementary Table 3 provides an overview of the characteristics of the WDZ at the source, treatment and zone level. About 45% of all WDZ are supplied by groundwater, 45% by surface water, and the remainder by both types of sources. Supplies using surface water alone serve over 60% of the total population. Protozoa log reduction is a variable used to indicate whether additional treatment is required based on the potential contamination of source waters. In total 334 WDZ serving 2.3 million people fall in the 99.99% reduction category which is typically assigned to sources located in an intensively farmed area. At a treatment level, only 8.9% of the population is on a WDZ without any protozoa barrier. Similarly, there is almost universal adoption of bacterial barriers (95.5% of the population), chlorination (94.8%) and residual disinfectant (96.3%), while 98.8% of the total population are on a supply categorised as large.

| **Water supply characteristics** | | **Number of WDZ*** | | **Population served on those WDZ** | |
| --- | --- | --- | --- | --- | --- |
|  | | **n** | **%** | **n** | **%** |
| **Source-level** | | | | | |
| **Source water type** | *Ground only* | 280 | 44.6 | 928,599 | 22.2 |
|  | *Surface only* | 285 | 45.4 | 2,602,065 | 62.3 |
|  | *Mixed* | 59 | 9.4 | 643,542 | 15.4 |
|  | *missing* | 4 | <1.0 | 1,247 | <1.0 |
| ***Protozoa log reduction*** | *0* | 39 | 6.2 | 301,636 | 7.2 |
|  | 99.9% | 216 | 34.4 | 1,487,899 | 35.6 |
|  | 99.99% | 334 | 53.2 | 2,371,456 | 56.8 |
|  | missing | 39 | 6.2 | 14,462 | <1.0 |
| **Treatment-level** | | | | | |
| **Protozoa barrier** | No | 122 | 19.4 | 371,533 | 8.9 |
|  | Yes | 472 | 75.2 | 3,382,520 | 81.0 |
|  | Mixed | 29 | 4.6 | 420,016 | 10.1 |
|  | missing | 5 | 0.8 | 1,384 | <1.0 |
| **Bacteria barrier** | No | 17 | 2.7 | 2,756 | 0.1 |
|  | Yes | 596 | 94.9 | 3,987,138 | 95.5 |
|  | Mixed | 10 | 1.6 | 184,175 | 4.4 |
|  | missing | 5 | 0.8 | 1,384 | <1.0 |
| **Ultraviolet light** | No | 266 | 42.4 | 2,538,553 | 60.8 |
|  | Yes | 315 | 50.2 | 1,033,167 | 24.7 |
|  | Mixed | 42 | 6.7 | 602,349 | 14.4 |
|  | missing | 5 | 0.8 | 1,384 | <1.0 |
| **Chlorination** | No | 23 | 3.7 | 30,625 | 0.7 |
|  | Yes | 590 | 93.9 | 3,959,269 | 94.8 |
|  | Mixed | 10 | 1.6 | 184,175 | 4.4 |
|  | missing | 5 | 0.8 | 1,384 | 0.0 |
| **Filtration** | No | 244 | 38.9 | 850,551 | 20.4 |
|  | Yes | 335 | 53.3 | 2,749,803 | 65.9 |
|  | Mixed | 44 | 7.0 | 573,715 | 13.7 |
|  | missing | 5 | 0.8 | 1,384 | 0.0 |
| **Distribution-level** | | | | | |
| **Supply size** | <50 people | 48 | 7.6 | 1,492 | 0.0 |
|  | 50-500 people | 211 | 33.6 | 50,564 | 1.2 |
|  | >500 people | 368 | 58.6 | 4,123,397 | 98.8 |
|  | missing | 1 | 0.2 | 0 | 0.0 |
| **Residual disinfectant** | No | 22 | 3.5 | 94,047 | 2.3 |
|  | Yes | 590 | 93.9 | 4,022,279 | 96.3 |
|  | Mixed | 16 | 2.5 | 59,127 | 1.4 |

*WDZ = Water Distribution Zone

# Supplementary Table 4. Drinking water data completeness by determinant in case period, case and any control period, and in the selected case and control periods.

| **Direct indicator** | **With case data** | **With case and any control** | **With case, control 1 and control 3** |
| --- | --- | --- | --- |
| E. coli | 30,362 | 30,337 | 29,854 |
| Total coliforms | 27,755 | 27,706 | 27,162 |
| FAC | 14,081 | 14,000 | 13,462 |
| Turbidity | 25,570 | 25,364 | 24,471 |
| pH | 23,822 | 23,511 | 22,931 |
| Temperature | 16,871 | 16,501 | 14,525 |
| Every direct indicator | 4,400 | 4,200 | 3,622 |

# Supplementary Table 5. Association between maximum daily ambient temperature and mean daily rainfall and enteric disease.

We identified significant associations between mean daily rainfall and enteric (OR 1.010, p=0.001) and bacterial diseases (OR 1.011, p=0.001) but not protozoan diseases (Supplementary Table 5). There was no significant association identified between any type of disease and the mean of maximum daily ambient temperature.

| **Weather variable** | **All enteric** | | **All bacterial** | | **All protozoan** | |
| --- | --- | --- | --- | --- | --- | --- |
|  | **OR** | **(95%CI)** | **OR** | **(95%CI)** | **OR** | **(95%CI)** |
| Mean of maximum daily ambient temperature | 0.999 | (0.998, 1.000) | 0.999 | (0.998, 1.000) | 1.001 | (0.998, 1.004) |
| Mean daily rainfall | **1.010** | **(1.004, 1.017)** | **1.011** | **(1.005, 1.018)** | 1.003 | (0.985, 1.022) |

*For all water sources, average rainfall by tertile was 0.7, 2.4, 5.8 mm/day and for sources requiring 99.99% reduction of protozoa, 0.8, 2.5, 5.9 mm/day.

# Supplementary Table 6. Association between E. coli and enteric disease in separate models by water supply characteristics (cases with imputed onset dates removed)

| **Water supply characteristic** | **All bacterial** | | | **All protozoan** | | |
| --- | --- | --- | --- | --- | --- | --- |
|  | **OR** | **(95%CI)** | | **OR** | **(95%CI)** | |
| **Source type** |  |  |  |  |  |  |
| Ground | 0.69 | 0.46 | 1.04 | 1.50 | 0.34 | 6.70 |
| Surface | 1.24 | 0.99 | 1.54 | 0.83 | 0.44 | 1.57 |
| Mixed | 1.11 | 0.83 | 1.50 | 0.74 | 0.29 | 1.91 |
| **Protozoa barrier** |  |  |  |  |  |  |
| No barrier | 0.82 | 0.51 | 1.33 | 0.30 | 0.03 | 2.87 |
| Barrier present | 1.21 | 0.99 | 1.47 | 1.11 | 0.64 | 1.94 |
| Mixed | 0.93 | 0.66 | 1.32 | 0.35 | 0.09 | 1.35 |
| **Protozoa log reduction** |  |  |  |  |  |  |
| 0-log | 0.90 | 0.54 | 1.49 | - | - | - |
| 3-log (99.9%) | 0.91 | 0.70 | 1.19 | 0.61 | 0.29 | 1.27 |
| 4-log (99.99%) | **1.29** | **1.03** | **1.62** | 1.44 | 0.70 | 2.94 |

# Supplementary Table 7. Association between the presence of E. coli in water suppliers with known source water risks (4-log protozoa reduction required) by age in quintile groupings

| **Characteristic** | **Category** | **OR** | **(95%CI)** | |
| --- | --- | --- | --- | --- |
| **Tertile of Age** | *0–8* | 1.26 | 0.83 | 1.90 |
|  | *9–27* | **1.47** | **1.06** | **2.05** |
|  | *28-41* | 1.18 | 0.82 | 1.70 |
|  | *42-59* | 1.44 | 0.98 | 2.13 |
|  | *60+* | 1.16 | 0.82 | 1.65 |
